# Protective Effect of Tropisetron on Rodent Hepatic Injury after Trauma-Hemorrhagic Shock through P38 MAPK-Dependent Hemeoxygenase-1 Expression

**DOI:** 10.1371/journal.pone.0053203

**Published:** 2012-12-28

**Authors:** Fu-Chao Liu, Huang-Ping Yu, Tsong-Long Hwang, Yung-Fong Tsai

**Affiliations:** 1 Department of Anesthesiology, Chang Gung Memorial Hospital, Taoyuan, Taiwan; 2 College of Medicine, Chang Gung University, Taoyuan, Taiwan; 3 Graduate Institute of Natural Products, Chang Gung University, Taoyuan, Taiwan; 4 Graduate Institute of Clinical Medical Sciences, Chang Gung University, Taoyuan, Taiwan; Southern Medical University, China

## Abstract

Tropisetron can decrease inflammatory cell responses and alleviate organ damage caused by trauma-hemorrhage, but the mechanism of these effects remains unknown. The p38 mitogen-activated protein kinase/hemeoxygenase-1 (p38 MAPK/HO-1) pathway exerts anti-inflammatory effects on different tissues. The aim of this study was to investigate whether p38 MAPK/HO-1 plays any role in the tropisetron-mediated attenuation of hepatic injury after trauma-hemorrhage. Male Sprague-Dawley rats underwent trauma-hemorrhage (mean blood pressure maintained at approximately 35–40 mmHg for 90 min), followed by fluid resuscitation. During resuscitation, several treatment regimens were administered: four doses of tropisetron alone (0.1, 0.3, 1, 3 mg/kg body weight), or a single dose of tropisetron (1 mg/kg body weight) with and without a p38 MAPK inhibitor (SB-203580, 2 mg/kg body weight) or HO antagonist (chromium-mesoporphyrin, 2.5 mg/kg body weight). Various parameters were measured, and the animals were sacrificed at 24 h post-resuscitation. The results showed that trauma-hemorrhage increased the following parameters: plasma concentrations of aspartate (AST) and alanine aminotransferases (ALT), hepatic myeloperoxidase (MPO) activity, and levels of cytokine-induced neutrophil chemoattractant-1 and -3 (CINC-1 and CINC-3), intercellular adhesion molecule-1 (ICAM-1), interleukin-6 (IL-6), tumor necrosis factor-α (TNF-α), and macrophage inflammatory protein-1α (MIP-1α). These parameters were significantly improved in the tropisetron-treated rats subjected to trauma-hemorrhage. Tropisetron treatment also increased hepatic p38 MAPK and HO-1 expression compared with vehicle-treated trauma-hemorrhaged rats. Co-administration of SB-203580 or chromium-mesoporphyrin with tropisetron abolished the tropisetron-induced beneficial effects on the above parameters and hepatic injury. These results suggest that the protective effect of tropisetron administration on alleviation of hepatic injury after trauma-hemorrhage is likely mediated through p38 MAPK-dependent HO-1 expression.

## Introduction

The liver is a critical highly vascularized organ within the abdominal cavity, which is commonly injured in trauma patients. Massive blood loss following liver injury causes hemorrhagic shock and organ dysfunction [1], [2]. The extent of hepatic dysfunction reflects the severity of organ injury and is associated with patient morbidity and mortality [1], [3].

The p38 mitogen-activated protein kinase (MAPK) may regulate inflammatory responses in different cells by various stimuli [4], [5]. P38 MAPK also plays an important role in shock-induced hepatic, myocardial and intestinal injuries [4], [6], [7]. Previous studies have shown that the induction of HO-1 expression could protect against organ injury and deleterious pathophysiological conditions, such as endotoxemia, oxidative stress, ischemia-reperfusion, trauma-hemorrhage, and wound healing [8]–[11]. A growing body of evidence indicates that activation of p38 MAPK induces HO-1 expression [4], [5], which is known to play a protective role in many organs under various deleterious conditions, including trauma-hemorrhage [12]. Several reports have also shown that up-regulation of HO-1 can attenuate organ injury during shock status through a decrease in cytokines, chemokines, and adhesion molecules and a reduction in neutrophil accumulation [10], [13].

Trauma-hemorrhage results in massive production of pro-inflammatory cytokines and chemokines [14]. Following trauma-hemorrhage, neutrophil migration and movement are mediated by pro-inflammatory mediators, as is their interaction with multiple adhesion molecules [15]–[17]. Pro-inflammatory chemokines, such as cytokine-induced neutrophil chemoattractant-1 and -3 (CINC-1 and CINC-3), are potent chemoattractants for neutrophils [6], [15]. Intercellular adhesion molecule-1 (ICAM-1) is up-regulated after trauma-hemorrhage, and it enhances a firm adhesion of neutrophils to the vascular endothelium [12], [18]. Interleukin-6 (IL-6), tumor necrosis factor-α (TNF-α), and macrophage inflammatory protein-1α (MIP-1α) also play *important role* in *neutrophil* infiltration and hepatic inflammation following organ injury [6], [18], [19].

Tropisetron, a 5-HT3 receptor inhibitor, has been reported to modulate various cells involved in immune response, and possess anti-phlogistic and anti-inflammatory activity [20], [21]. Previous studies have shown that tropisetron exhibits neuroprotective activity in a rat ischemic brain injury model [22]. Our recent studies have also shown that tropisetron can attenuate cardiac injury after trauma-hemorrhage through attenuation of pro-inflammatory mediator production [23]. However, it remains unknown whether p38 MAPK/HO-1 plays a critical role in the tropisetron-mediated organ protection after trauma-hemorrhage. Therefore, we hypothesized that the protective effects of tropisetron after trauma-hemorrhage are mediated via the p38 MAPK-dependent HO-1 pathway. To test this hypothesis, animals were treated with tropisetron alone and in combination with a p38 MAPK inhibitor SB-203580 or the HO antagonist chromium-mesoporphyrin after trauma-hemorrhage. After trauma-hemorrhage, the effects of these treatments were then examined on plasma aspartate aminotransferase (AST) and alanine aminotransferase (ALT) levels, and hepatic tissue myeloperoxidase (MPO) activity and the levels of chemokine (CINC-1 and CINC-3), ICAM-1, IL-6, TNF-α, and MIP-1α and p38 MAPK/HO-1 expression were evaluated.

## Materials and Methods

### Animals

Adult male Sprague-Dawley strain rats were used in this study. The rats were obtained from the National Science Council Experimental Animal Center. All animal experiments were performed according to the guidelines of the *Animal Welfare Act* and *The Guide for Care and Use of Laboratory Animals* from the National Institutes of Health. All procedures and protocols were approved by the Institutional Animal Care and Use Committee of Chang Gung Memorial Hospital.

### Experimental Groups

Male Sprague-Dawley rats (285–325 g) were used for evaluation of the dose-response relationship of tropisetron on hepatic injury after trauma-hemorrhage. Thirty-six rats were randomly assigned to 6 groups (n = 6/group). Initial studies examined trauma-hemorrhage, with the groups receiving tropisetron (0, 0.1, 0.3, 1, or 3 mg/kg); sham groups were also included. In addition, sixty-four male Sprague-Dawley rats were randomly divided into 8 separate groups (n = 8/group). These trauma-hemorrhage rats were treated with tropisetron (1 mg/kg), tropisetron co-administered with a specific p38 MAPK inhibitor (SB-203580∶2 mg/kg), SB-203580 alone, tropisetron (1 mg/kg) co-administered with the HO inhibitor chromium-mesoporphyrin (2.5 mg/kg), chromium-mesoporphyrin alone, or an equal volume of the vehicle. Sham groups were also included.

### Rat Trauma-Hemorrhagic Shock Model

Male Sprague-Dawley strain rats were used in this study. All animals were housed individually in cages with air-conditioning (humidity 70–75%), temperature 24–25°C, and controlled lighting (light-dark cycles every 12 h: lights on 06∶00 to 18∶00). Basal diet and water were provided, and the animals were allowed at least 1 wk to adapt to the environment. Before the onset of the experiment, the rats were fasted overnight but allowed free water access. Trauma-hemorrhagic shock and resuscitation were then performed as described previously [15]. Briefly, the rats were anesthetized by isoflurane inhalation, and a 5-cm midline laparotomy was performed to induce soft tissue trauma, then the abdominal wound was closed in layers. Polyethylene catheters (PE-50; Becton Dickinson & Co., Sparks, MD) were placed in both femoral arteries and the right femoral vein from bilateral inguinal incision wounds (about 0.5 cm in length), and the bilateral inguinal incision sites were then closed. The wounds were bathed with 1% lidocaine (Elkins-Sinn Inc., Cherry Hill, NJ) throughout the operative procedure to reduce postoperative pain. The rats were allowed to awaken, after which they were bled rapidly within 10 minutes to a mean arterial pressure of 35 to 40 mmHg. This level of hypotension was maintained until the animals could no longer maintain a mean arterial pressure of 40 mmHg unless some fluid in the form of Ringer’s lactate was administered. This time was defined as maximum bleed-out. After the maximal bleed-out, mean arterial pressure was maintained between 35 to 40 mmHg until 40% of the maximal bleed-out volume was returned in the form of Ringer’s lactate solution (about 90 min from the onset of bleeding). The rats were then resuscitated with four times the volume of the shed blood with Ringer’s lactate for 60 min. Thirty minutes before the end of the resuscitation period, the rats received tropisetron alone (0.1, 0.3, 1 or 3 mg/kg intravenously), a combination of tropisetron (1 mg/kg) plus the specific p38 MAPK inhibitor SB-203580 (2 mg/kg, intravenously at the beginning of resuscitation), combination of tropisetron plus the HO inhibitor chromium-mesoporphyrin (2.5 mg/kg, intraperitoneally at the beginning of resuscitation), or an equal volume of the vehicle (about 0.2 mL, 10% DMSO). After resuscitation, the catheters were removed, the vessels were ligated, and the skin incisions were closed with sutures. Sham-operated animals did not undergo laparotomy. In addition, neither hemorrhage nor resuscitation was performed for sham-operated animals. Vehicle or tropisetron were also administered in sham-operated rats after catheters were placed. The animals were humanely sacrificed at 24 h after the end of the resuscitation or sham operation. In this group of experiments, there were 8 animals in each group.

### Measurement of Hepatic Injury

At 24 h after trauma-hemorrhage or sham operation, blood samples were obtained with heparin, and plasma was separated by centrifugation. Hepatic injury was determined by measuring plasma levels of AST and ALT using a colorimetric analyzer (Dri-Chem 3000; Fuji Photo Film Co., Tokyo, Japan).

### Measurement of Myeloperoxidase Activity

MPO activity in homogenates of whole liver was determined as described previously [15]. Frozen tissue samples were thawed and suspended in phosphate buffer (pH 6.0) containing 0.5% hexadecyltrimethylammonium bromide (Sigma, St. Louis, MO). The samples were sonicated on ice, centrifuged at 12,000 g for 15 minutes at 4°C, and an aliquot was transferred into phosphate buffer (pH 6.0) containing 0.167 mg/mL o-dianisidine hydrochloride and 0.0005% hydrogen peroxide (Sigma). The change in absorbance at 460 nm was measured spectrophotometrically for 5 minutes. MPO activity was calculated using a standard curve that was generated using human MPO (Sigma), and values were normalized to protein concentration.

### Determination of CINC-1, CINC-3, ICAM-1, IL-6, TNF-α, and MIP-1α Levels

The liver tissues were homogenized in PBS (1∶10 weight:volume; pH 7.4) containing protease inhibitors (Complete Protease Inhibitor Cocktail; Boehringer, Mannheim, Germany). The homogenates were centrifuged at 2,000 g for 20 minutes at 4°C, and the supernatant were analyzed for the presence of CINC-1, CINC-3, ICAM-1, IL-6, and TNF-α using ELISA kits (R&D, Minneapolis, MN) and MIP-1α using ELISA kit (Koma Biotech, Seoul, Korea), according to the manufacturer’s instructions and as described previously [15]. An aliquot of the supernatant was used to determine protein concentration by the Bio-Rad DC Protein Assay (Bio-Rad, Hercules, CA).

### Western Blot Assay

Protein aliquots were used to determine protein concentration with the Bio-Rad DC Protein Assay (Bio-Rad, Hercules, CA). Samples were mixed with 4× sample buffer, electrophoresed on sodium dodecyl sulfate-polyacrylamide gels and transferred electrophoretically onto nitrocellulose paper. The membranes were then incubated overnight at 4°C with antibodies against total p38 MAPK protein (1∶2000 dilution; Cell Signaling Technology Inc., Beverly, MA), phospho (p)-p38 MAPK (1∶1000 dilution; Cell Signaling Technology Inc., Beverly, MA), the HO-1 protein (1∶6000 dilution; Chemicon International, Temecula, CA), and GAPDH (1∶15000 dilution; Abcam, Cambridge, MA). The membranes were then incubated with horseradish peroxidase-conjugated goat anti-rabbit antibody or goat anti-mouse antibody for 1.5 h at room temperature. After the final wash, the membranes were probed using enhanced chemiluminescence (Amersham, Piscataway, NJ) and exposed to film.

### Histological Examination of the Liver

Examination of liver histology, three pieces of the middle lobe were fixed in 10% formalin in phosphate-buffered saline for 24 h and were sent to the histology laboratory for further processing. Sections were embedded in paraffin and then cut (4–5 µM) and mounted on glass slides. Liver sections were stained with hematoxylin-eosin, observed under the microscope (Nikon Eclipse TS100) at 200x magnification for changes in liver morphology, and photographed (SPOT, RTcolor, Diagnostic Instrument, Inc., Iowa City, Iowa) using a microscope-attached camera.

### Statistical Analysis

For statistical analysis we used the InStat 3.0 biostatistics program (Graph Pad Software Inc., San Diego, CA). Results are presented as the mean ± standard error of the mean (SEM). The data were analyzed using one-way analysis of variance (ANOVA) and the Tukey test. Differences were considered significant at *p*<0.05.

## Results

### Dose-Response Effects of Tropisetron on Plasma AST and ALT Levels

**Figure 1:**
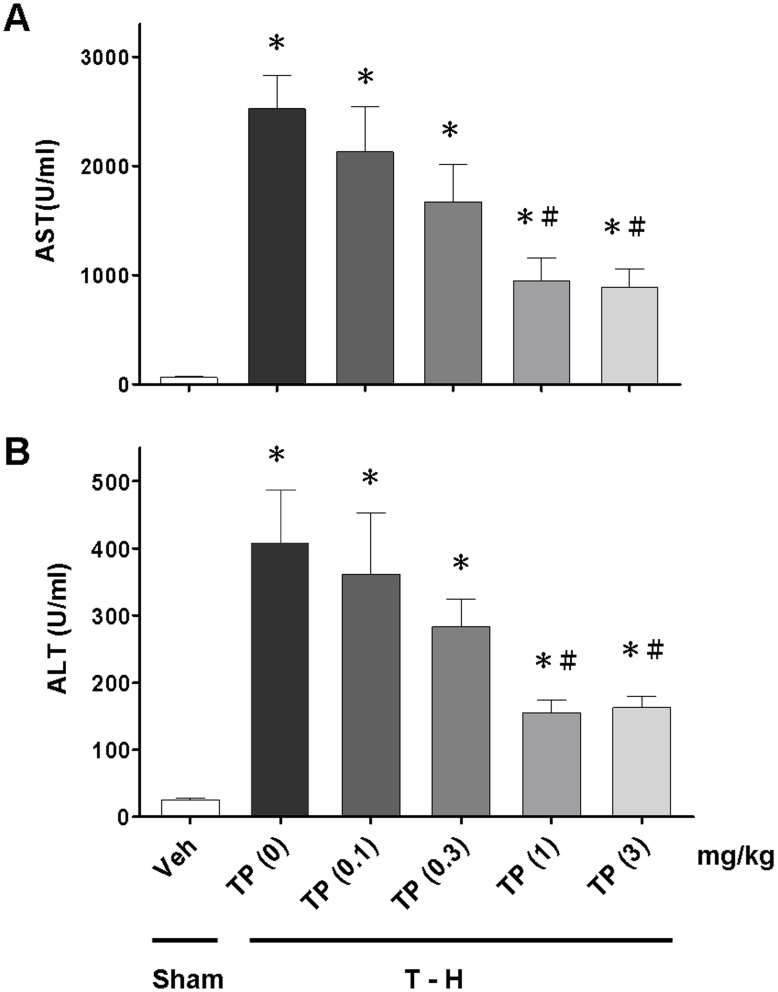
Dose-dependent responses to tropisetron treatment of plasma AST (A) and ALT (B) in rats at 24 h after sham operation (sham) or trauma-hemorrhage and resuscitation (T–H). Animals were treated with tropisetron (TP) at doses of 0, 0.1, 0.3, 1 or 3 mg/kg. Data are shown as the mean ± SEM. n = 6 rats in each group. *p<0.05 compared with sham; ^#^ *p*<0.05 compared with T–H+TP (0 mg/kg).

As shown in Figures 1A and 1B, trauma-hemorrhage was related to a significant increase in plasma AST and ALT levels at 24 h after resuscitation. Administration of tropisetron at a dose of 0.1, 0.3, 1, or 3 mg/kg was used to evaluate the effects of tropisetron on the attenuation of hepatic injury after trauma-hemorrhage. As shown in Figure 1, there was a diminished benefit when tropisetron was administered at the dose of 0.1 or 0.3 mg/kg. The effects of tropisetron were equivalent when administered at a dose of 1 or 3 mg/kg.

### Alteration of Plasma AST and ALT Levels

**Figure 2:**
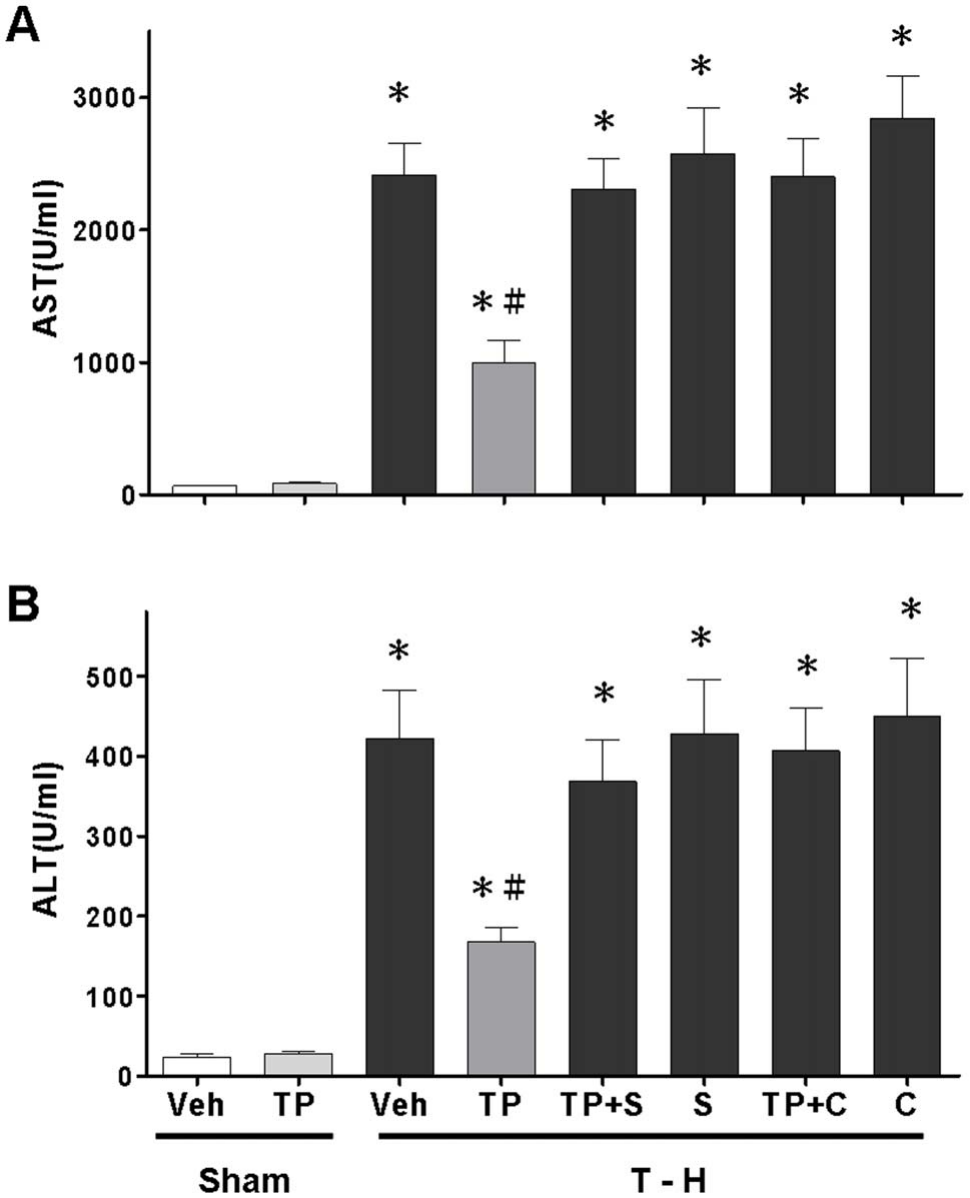
Effect of tropisetron treatment on plasma AST (A) and ALT (B) in rats after sham operation (Sham) or trauma-hemorrhage and resuscitation (T–H). Animals were treated with vehicle (Veh), tropisetron (TP), tropisetron in combination with SB-203580 (TP+S), SB-203580 (S), tropisetron in combination with chromium-mesoporphyrin (TP+C), or chromium-mesoporphyrin (C). Data are shown as the mean ± SEM; n = 8 rats in each group. *p<0.05 compared with sham; ^#^ *p*<0.05 compared with T–H+Veh, T–H+TP+S, T–H+S, T–H+TP+C, and T–H+C. (SB-203580: a p38 MAPK inhibitor and chromium-mesoporphyrin: a HO antagonist).

As shown in Figures 2A and 2B, no significant difference in plasma AST and ALT levels were observed between the vehicle- and tropisetron-treated sham groups. At 24 h after trauma-hemorrhage, there were significant increases in plasma AST and ALT levels. Tropisetron (1 mg/kg) treatment attenuated the trauma-hemorrhage-induced increase in plasma AST and ALT levels. To determine whether the salutary effects of tropisetron in attenuating hepatic injury after trauma-hemorrhage were mediated via a p38 MAPK-mediated activity, a group of tropisetron-treated trauma-hemorrhage rats were administered p38 MAPK inhibitor SB-203580. The results indicated that the administration of the p38 MAPK inhibitor SB-203580 prevented the tropisetron-induced decrease in plasma AST and ALT levels. To determine whether tropisetron reduced hepatic injury after trauma-hemorrhage via a HO-1-mediated pathway, a group of animals were administered HO enzyme inhibitor chromium-mesoporphyrin with tropisetron. The results indicated that administration of chromium-mesoporphyrin with tropisetron prevented the tropisetron-induced decrease in plasma AST and ALT levels.

### Alteration in Hepatic MPO Activity

**Figure 3:**
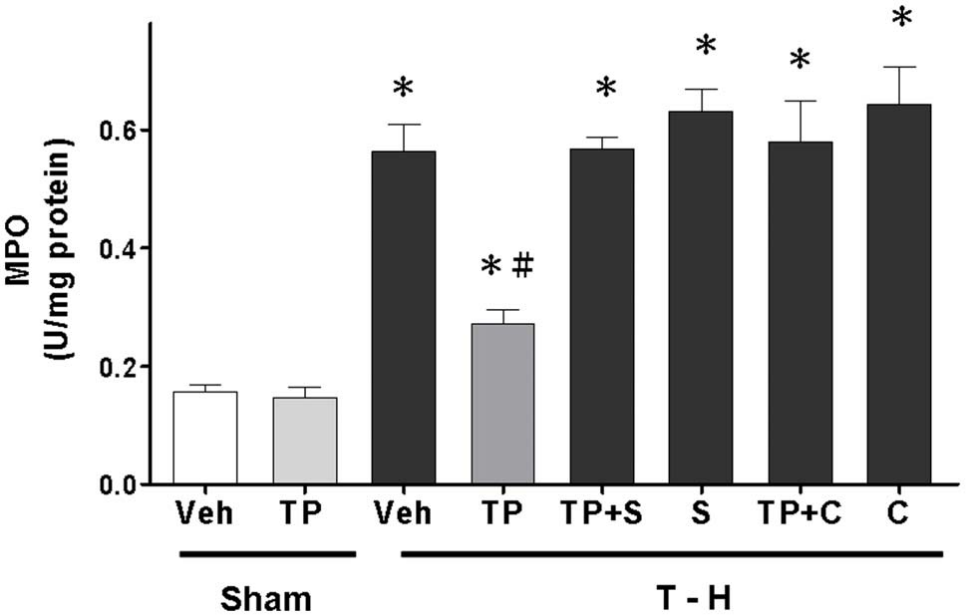
Effect of tropisetron treatment on hepatic MPO activity in rats after sham operation (Sham) or trauma-hemorrhage and resuscitation (T–H). Animals were treated with vehicle (Veh), tropisetron (TP), tropisetron in combination with SB-203580 (TP+S), SB-203580 (S), tropisetron in combination with chromium-mesoporphyrin (TP+C), or chromium-mesoporphyrin (C). Data are shown as the mean ± SEM; n = 8 rats in each group. *p<0.05 compared with sham; ^#^ *p*<0.05 compared with T–H+Veh, T–H+TP+S, T–H+S, T–H+TP+C, and T–H+C.

These studies showed that there were no differences in hepatic MPO activity between the vehicle- and tropisetron-treated sham groups (Figure 3). After trauma-hemorrhage, MPO activity was significantly increased in vehicle-treated rats compared with sham-operated animals. Tropisetron treatment attenuated this increase in hepatic MPO activity. Administration of the p38 MAPK inhibitor SB-203580 prevented the tropisetron-mediated attenuation of hepatic MPO activity after trauma-hemorrhage. To evaluate the role of HO-1 in the tropisetron-induced decrease in hepatic MPO activity in trauma-hemorrhaged rats, animals were treated with the HO inhibitor chromium-mesoporphyrin with tropisetron after trauma-hemorrhage. The results indicated that administration of chromium-mesoporphyrin with tropisetron prevented the tropisetron-induced decrease in hepatic MPO activity.

### Alteration of Hepatic CINC-1, CINC-3, and ICAM-1 Expression

**Figure 4:**
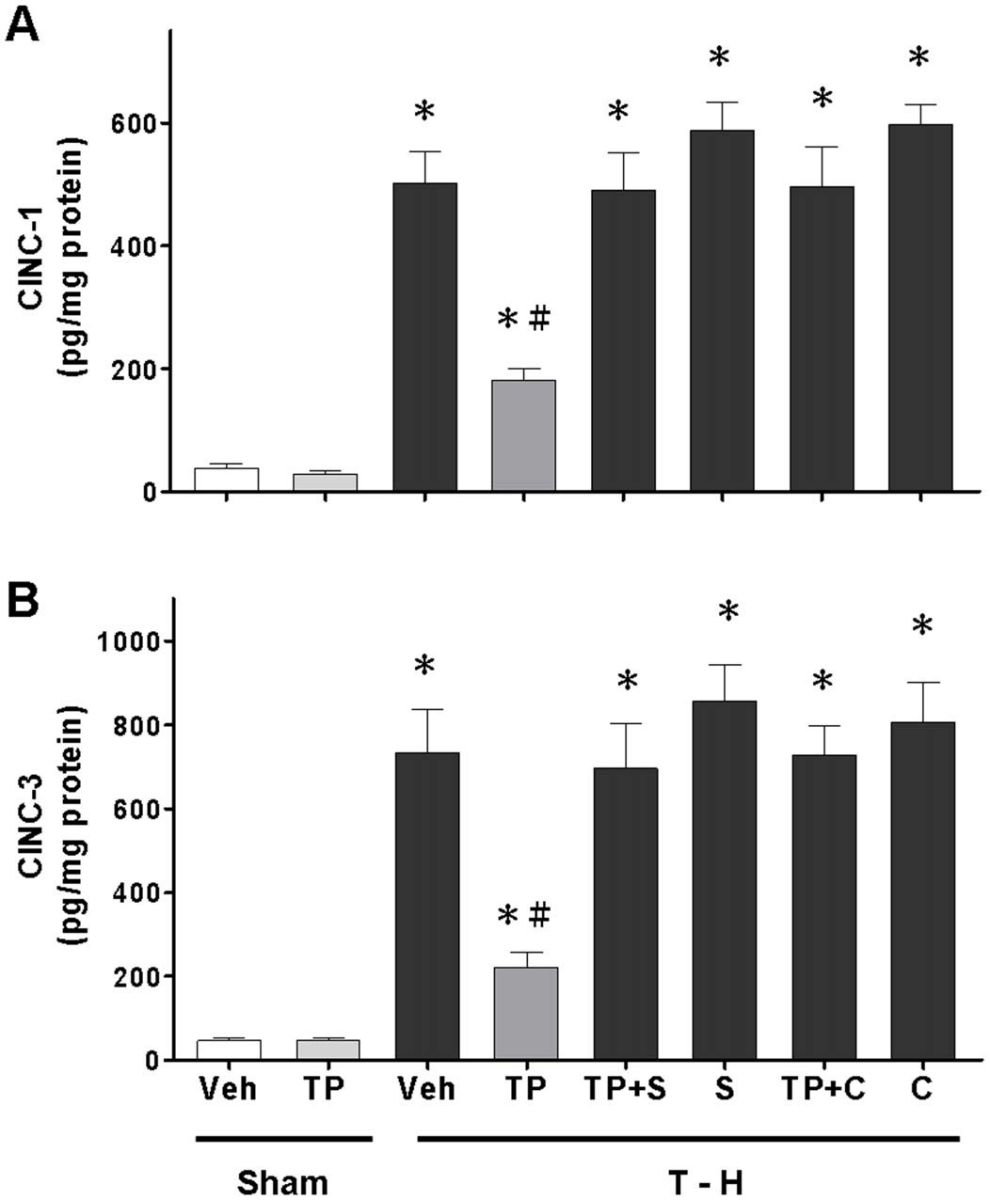
Effect of tropisetron treatment on hepatic CINC- 1 (A) and CINC-3 (B) levels in rats after sham operation (Sham) or trauma-hemorrhage and resuscitation (T–H). Animals were treated with vehicle (Veh), tropisetron (TP), tropisetron in combination with SB-203580 (TP+S), SB-203580 (S), tropisetron in combination with chromium-mesoporphyrin (TP+C), or chromium-mesoporphyrin (C). Data are shown as the mean ± SEM; n = 8 rats in each group. *p<0.05 compared with sham; ^#^ *p*<0.05 compared with T–H+Veh, T–H+TP+S, T–H+S, T–H+TP+C, and T–H+C.

**Figure 5:**
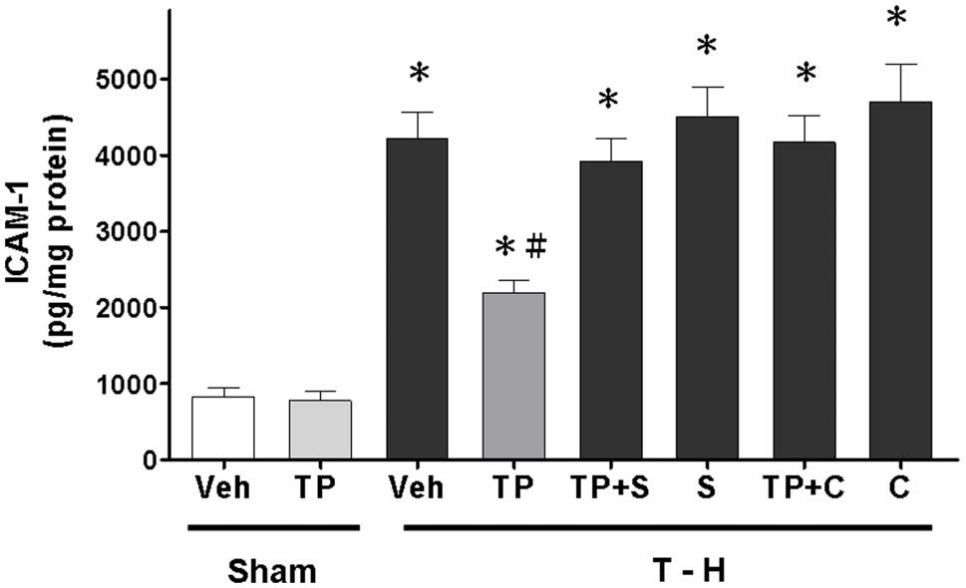
Effect of tropisetron treatment on hepatic ICAM-1 levels in rats after sham operation (Sham) or trauma-hemorrhage and resuscitation (T–H). Animals were treated with vehicle (Veh), tropisetron (TP), tropisetron in combination with SB-203580 (TP+S), SB-203580 (S), tropisetron in combination with chromium-mesoporphyrin (TP+C), or chromium-mesoporphyrin (C). Data are shown as the mean ± SEM; n = 8 rats in each group. *p<0.05 compared with sham; ^#^ *p*<0.05 compared with T–H+Veh, T–H+TP+S, T–H+S, T–H+TP+C, and T–H+C.

Trauma-hemorrhage significantly increased CINC-1, CINC-3, and ICAM-1 expression in the liver (Figures 4A, 4B, and 5). Treatment with tropisetron attenuated the trauma-hemorrhage-induced increase in CINC-1, CINC-3, and ICAM-1 expression. Co-administration of the p38 MAPK inhibitor SB-203580 with tropisetron prevented the tropisetron-induced reduction in CINC-1, CINC-3, and ICAM-1 expression. Moreover, co-administration of the HO inhibitor chromium-mesoporphyrin with tropisetron prevented the tropisetron-induced reduction in CINC-1, CINC-3, and ICAM-1 expression in the trauma-hemorrhaged group.

### Alteration of Hepatic IL-6, TNF-α, and MIP-1α Levels

**Figure 6:**
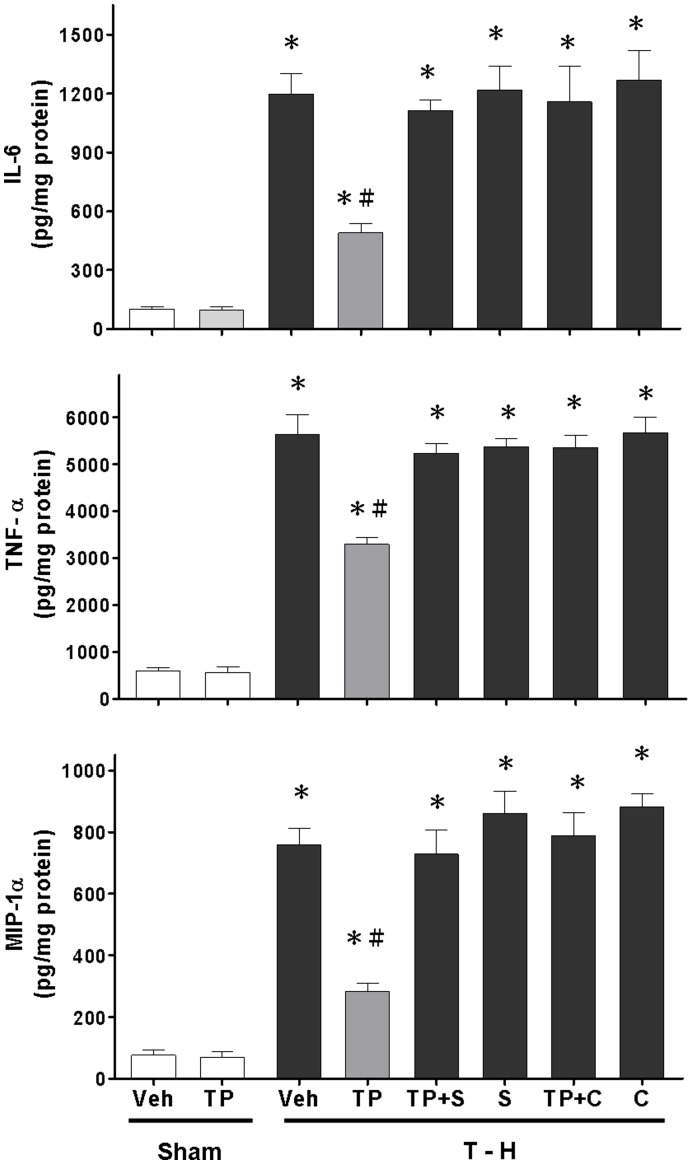
Effect of tropisetron treatment on hepatic IL-6 (A), TNF-α (B), and MIP-1α (C) levels in rats after sham operation (Sham) or trauma-hemorrhage and resuscitation (T–H). Animals were treated with vehicle (Veh), tropisetron (TP), tropisetron in combination with SB-203580 (TP+S), SB-203580 (S), tropisetron in combination with chromium-mesoporphyrin (TP+C), or chromium-mesoporphyrin (C). Data are shown as the mean ± SEM; n = 8 rats in each group. *p<0.05 compared with sham; ^#^ *p*<0.05 compared with T–H+Veh, T–H+TP+S, T–H+S, T–H+TP+C, and T–H+C.

There was no significant difference in hepatic IL-6, TNF-α, and MIP-1α levels between the vehicle- and tropisetron-treated sham groups (Figures 6A, 6B, and 6C). Trauma-hemorrhage significantly increased hepatic IL-6, TNF-α, and MIP-1α levels in vehicle-treated rats compared with sham animals. The increase in hepatic IL-6, TNF-α, and MIP-1α levels was reduced by tropisetron treatment, and the tropisetron-mediated reduction in IL-6 levels was abolished by SB-203580 co-administration. Furthermore, the tropisetron-mediated reduction in hepatic IL-6, TNF-α, and MIP-1α levels were also abolished by chromium-mesoporphyrin co-administration.

### P38 MAPK Phosphorylation, Protein Expression, and Activity

**Figure 7:**
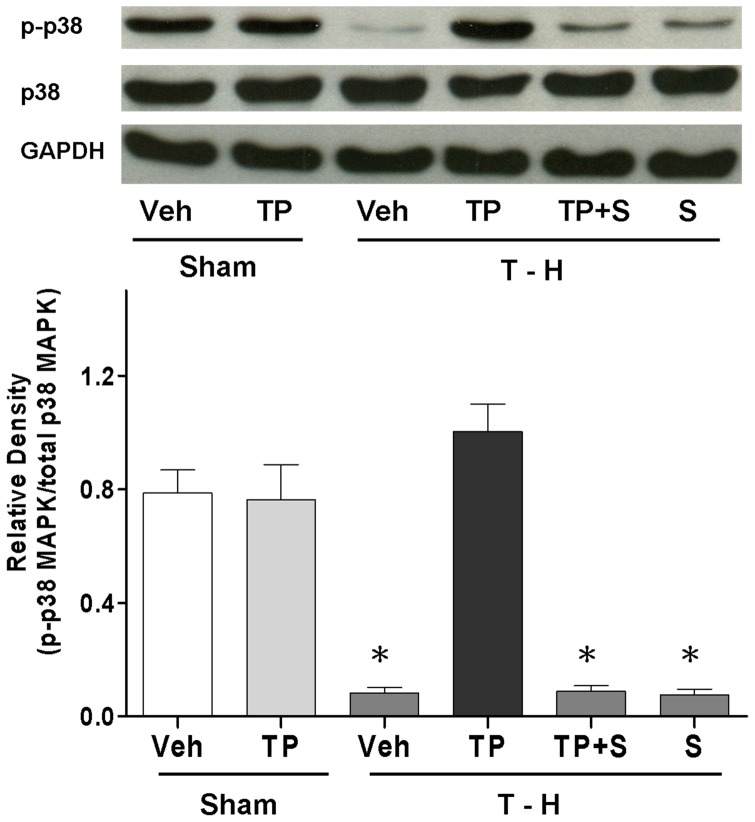
Hepatic p-p38 MAPK and p38 MAPK protein expression from sham animals receiving vehicle (Sham+Veh; lane 1) or tropisetron (Sham+TP; lane 2), trauma-hemorrhage receiving vehicle (T–H+Veh; lane 3), tropisetron (T–H+TP; lane 4), tropisetron and SB-203580 (T–H+TP+S; lane 5) or SB-203580 (T–H+S; lane 6). Blots were reprobed for GAPDH to determine equal protein loading in the various lanes. The bands were analyzed using densitometry, and the values are presented as the mean ± SEM; n = 8 rats per group. *p<0.05 versus sham and T–H+TP.

There was no significant difference in p38 MAPK protein expression between the sham and trauma-hemorrhaged rats (Figure 7). However, p38 MAPK activity as determined by its phosphorylation was significantly decreased after trauma-hemorrhage. Administration of tropisetron after trauma-hemorrhage restored p38 MAPK activity to the levels observed in the sham animals. The increase in phosphorylated-p38 MAPK induced by tropisetron was abolished when SB-203580 was administered along with tropisetron (Figure 7).

### HO-1 Expression in the Liver

**Figure 8:**
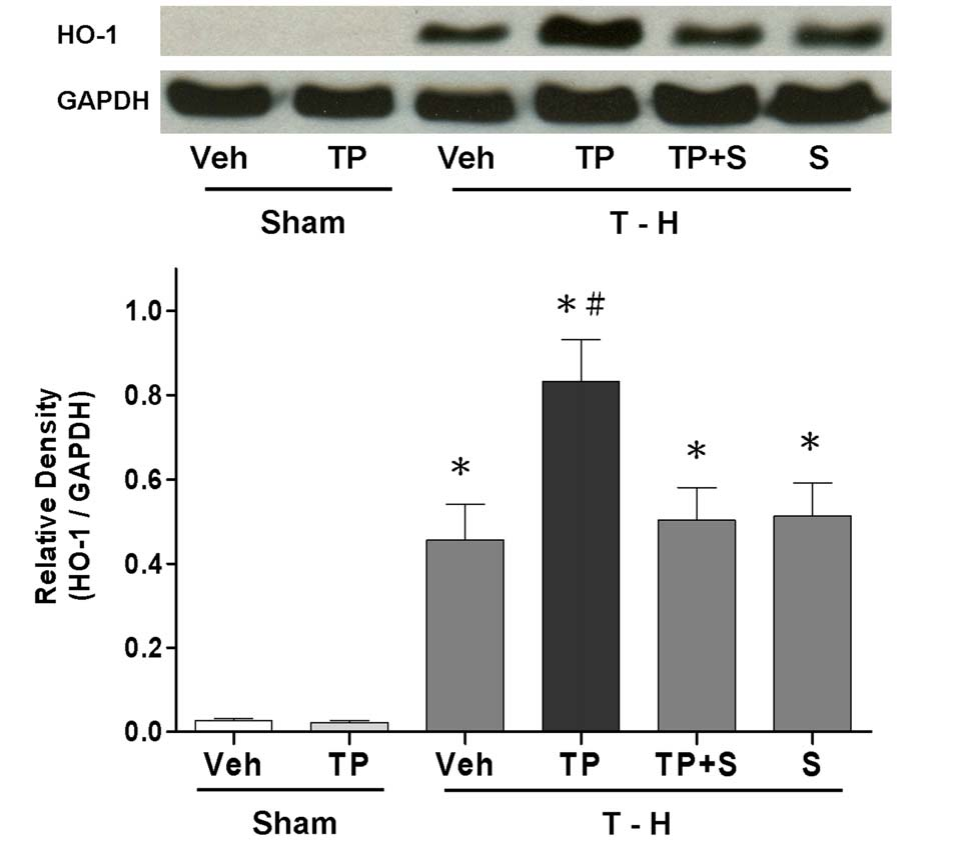
Hepatic HO-1 protein expression from sham animals receiving vehicle (Sham+Veh; lane 1) or tropisetron (Sham+TP; lane 2), trauma-hemorrhage receiving vehicle (T–H+Veh; lane 3), tropisetron (T–H+TP; lane 4), tropisetron and SB-203580 (T–H+TP+S; lane 5), or SB-203580 (T–H+S; lane 6). To determine equal protein loading, membranes were reprobed for GAPDH using a mouse monoclonal antibody. The bands were analyzed using densitometry, and the values are presented as the mean ± SEM; n = 8 rats per group. *p<0.05 compared with sham; ^#^ *p*<0.05 compared with T–H+Veh, T–H+TP+S, and T–H+S.

Trauma-hemorrhage induced a significant increase in hepatic HO-1 protein expression compared with its expression in shams animals (Figure 8). Administration of tropisetron after trauma-hemorrhage induced a further significant increase in hepatic HO-1 protein expression compared with its expression in vehicle-treated trauma-hemorrhaged rats. Because tropisetron up-regulated hepatic HO-1 after trauma-hemorrhage, we examined whether administration of the p38 MAPK inhibitor SB-203580 had any effect on HO-1 protein levels. These experiments indicated that co-administration of SB-203580 prevented the tropisetron-induced up-regulation of hepatic HO-1 after trauma-hemorrhage (Figure 8).

### Histological Analysis of the Liver

**Figure 9:**
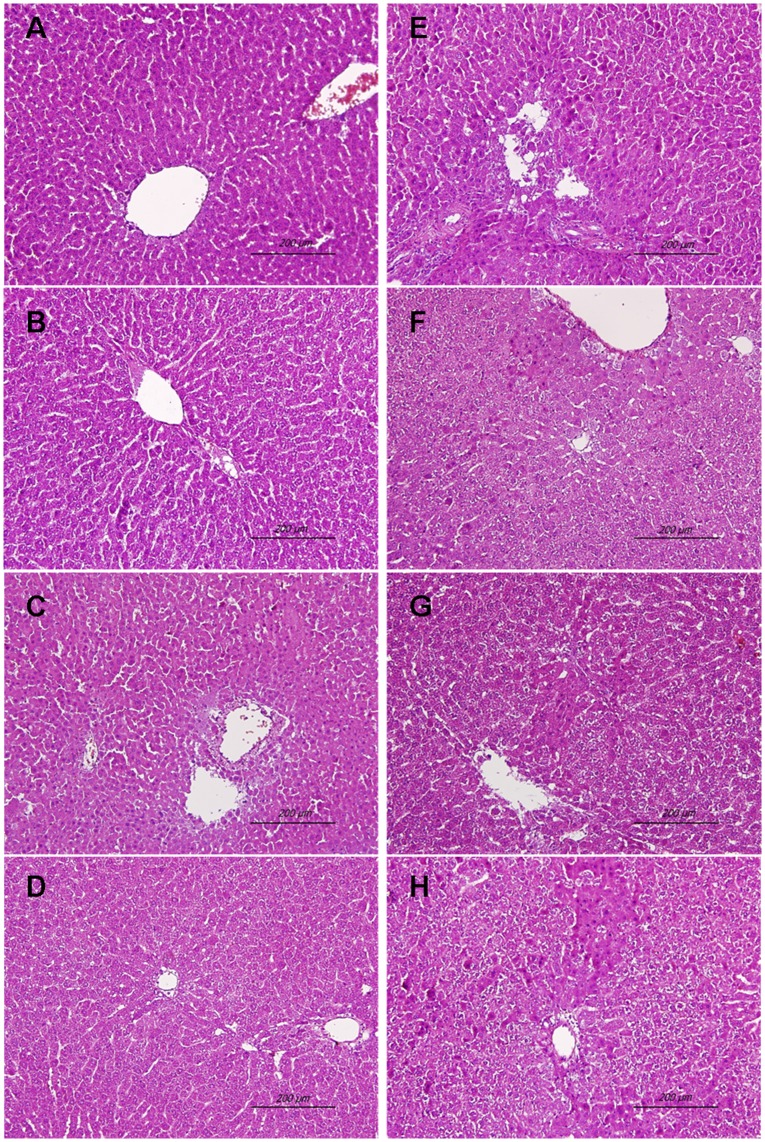
Representative histological photomicrographs of liver of sham animals receiving vehicle (A), sham animals receiving tropisetron (B), trauma-hemorrhage animals receiving vehicle (C), trauma-hemorrhage animals receiving tropisetron (D), trauma-hemorrhage animals receiving tropisetron and SB-203580 (E), trauma-hemorrhage animals receiving SB-203580, (F), trauma-hemorrhage animals receiving tropisetron and chromium-mesoporphyrin (G), and trauma-hemorrhage animals receiving chromium-mesoporphyrin (H). Animals were killed 24 h after sham operation or trauma-hemorrhage with resuscitation. Liver were removed and processed as described in the Methods. Liver sections were stained with hematoxylin-eosin, examined at an original magnification X200, and photographed.

Representative photomicrographs of liver are presented for sham animals treated with vehicle (Figure 9A), sham animals treated with tropisetron (Figure 9B), trauma-hemorrhage animals treated with vehicle (Figure 9C), trauma-hemorrhage animals treated with tropisetron (Figure 9D), trauma-hemorrhage animals receiving tropisetron and SB-203580 (Figure 9E), trauma-hemorrhage animals receiving SB-203580 (Figure 9F), trauma-hemorrhage animals receiving tropisetron and chromium-mesoporphyrin (Figure 9G), and trauma-hemorrhage animals receiving chromium-mesoporphyrin (Figure 9H). Similar results were obtained from four or more animals in each group. Together, these results, as presented in Figure 9, suggested that tropisetron ameliorated trauma-hemorrhage-induced damage in the liver.

## Discussion

In this study, we sought to determine whether p38MAPK-dependent HO-1 expression plays a critical role in the tropisetron-mediated hepatoprotection after trauma-hemorrhage. The present results indicated that at 24 h after trauma-hemorrhage, plasma AST and ALT concentrations, hepatic MPO activity, and CINC-1, CINC-3, ICAM-1, IL-6, TNF-α, and MIP-1α levels were all markedly increased in male rats. The salutary effects of tropisetron at doses of 0.1, 0.3, 1, and 3 mg/kg were evaluated for liver injury after trauma-hemorrhage. We found similar beneficial effects when tropisetron was administered at dosages of 1 or 3 mg/kg and poor results at dosages of 0.1 or 0.3 mg/kg. Administration of a single dose of tropisetron (1 mg/kg) during resuscitation could attenuate the increase in inflammatory parameters. Tropisetron administration also prevented the trauma-hemorrhage-induced decrease in hepatic p-p38 MAPK expression. A difference in the magnitude of HO-1 expression in the livers of male rats, with or without tropisetron treatment after trauma-hemorrhage, was also found. Co-administration of the p38 MAPK inhibitor (SB-203580) or HO inhibitor (chromium-mesoporphyrin) with tropisetron after trauma-hemorrhage abolished the tropisetron-induced effects described above. These results collectively suggest that the salutary effects of tropisetron are mediated via p38MAPK-dependent HO-1 expression.

The liver is a large solid abdominal organ, and liver damage can result in serious inflammation and life threatening conditions. Previous studies have shown that hepatic injury is associated with increased neutrophil accumulation [24]. The infiltration of neutrophils in the liver is also accompanied by increased expression of local cytokines, chemokines, and adhesion molecules [24]–[26]. IL-6 is an important pro-inflammatory mediator in hepatic damage and is required for chemokine production and adhesion molecule expression. Liver injury or hypoxia causes marked increases in hepatic IL-6 expression [18], [26]. In this study, hepatic IL-6, TNF-α, and MIP-1α levels were significantly attenuated in the animals treated with tropisetron after trauma-hemorrhage. Tissue MPO activity is an important indicator of neutrophil infiltration, and it has been correlated with CINC-1 and CINC-3 expression after trauma-hemorrhage [12], [18]. Our results showed that trauma-hemorrhage results in a significant increase in hepatic cytokine/chemokine levels and ICAM-1 expression, which are accompanied by elevated hepatic MPO activity. However, tropisetron administration after trauma-hemorrhage attenuated these pro-inflammatory mediators and alleviated hepatic injury.

Our previous studies have shown that the p38 MAPK pathway attenuates overproduction of pro-inflammatory cytokines, chemokines, and adhesion molecules and neutrophil accumulation after trauma-hemorrhage [6], [12]. P38 MAPK phosphorylation has been reported to activate protective effects in various organs, including the intestine, liver, and heart, after trauma-hemorrhage [4], [7], [12], [27]. In this study, we found that the tropisetron-induced attenuation of hepatic injury was likely mediated by increases in p38 MAPK activation, which was blocked by the p38 MAPK inhibitor SB-203580. Consistent with some studies, our results showed that trauma-hemorrhagic shock led to a significant decrease of p38 MAPK activation [4], [6], [7], [12], [27], [28]. However, this result has serious discrepancies with other reports [29]–[32]. Sato et al. have shown that hemorrhagic shock induces p38 MAPK activation in a male Wistar rat model of hemorrhagic shock without trauma or resuscitation [29], [31]. Li et al. indicate that hemorrhagic shock increases phosphorylation of p38γ MAPK, but decreases phosphorylation of p38α MAPK in a female C57BL6/J mice model of hemorrhagic shock/resuscitation without trauma [30]. Other study shows that hemorrhagic shock increases p38 MAPK expression in a male C57BL6/J mice model of hemorrhagic shock/resuscitation without trauma [32]. The animal model applied in our study is male Sprague-Dawley rat model of trauma-hemorrhagic shock with resuscitation. The mechanisms responsible for the divergent responses remain unknown. Previous studies have also shown that p38 MAPK activation contributes to the protection of cell/tissue following injury [5], [33], [34]. In human monocytes, the increase in p38 MAPK activation is associated with the alcohol-induced attenuation of TNF-α production and augmentation of IL-10 secretion [5]. Other reports also indicate that p38 MAPK activation protects glomerular epithelial cells against complement-mediated cell injury and regulates mucosal recovery in ischemic-injured ileum [33], [34]. The roles of p38 MAPK on tissue or organ function after injury might be complex. The possible reasons might be related to different species and various animal models among those studies. However, it remained to be determined in further studies.

Induction of HO-1 plays an important role in organ protection against oxidative stress, inflammation and biological toxicity [35]–[37]. A growing body of evidence indicates that HO-1 expression is up-regulated after trauma-hemorrhage and that its induction might play a critical role in the preservation of organ microcirculatory function and cytoprotection [4], [12]. Previous studies have shown that p38 MAPK activation leads to the induction of HO-1 [4], [5], [12]. Our results also suggest that the salutary effects of tropisetron are mediated via p38 MAPK-dependent HO-1 up-regulation. Our finding that treatment with SB-203580 abolished the tropisetron-induced up-regulation of HO-1 after trauma-hemorrhage suggests that tropisetron administration after trauma-hemorrhage up-regulates HO-1 via the p38 MAPK-related pathway. Our data showed that p38 MAPK activation might have organ protective effects in a rodent model of trauma-hemorrhagic shock. However, some reports indicate that p38 MAPK activation is harmful to organ functions in hemorrhagic shock [29]–[32]. The p38 MAPK activation might initiate the activation of HO-1 and have no direct organ protective effect. HO-1 might have direct organ protective effects. Further studies are required to elucidate the precise mechanism.

5-HT3 receptor antagonists are often used for prevention or treatment of nausea and vomiting [38], [39]. Previous studies have shown that tropisetron possesses anti-inflammatory properties [21], [22], which are related to the inhibition of pro-inflammatory mediator release from various tissues [40]. However, little is known about the role of tropisetron in trauma-hemorrhage. In the present study, tropisetron played a role in regulating the production of pro-inflammatory mediators. The ability of tropisetron to modulate expression of inflammatory cytokines, as well as chemokines and adhesion molecules, suggests a role for tropisetron in the regulation of hepatic inflammation. Our study further indicates that tropisetron administration after trauma-hemorrhage decreases pro-inflammatory mediator levels and likely attenuates liver injury through p38 MAPK-mediated up-regulation of HO-1.

From our study, tropisetron-induced organ protective effects are partially mediated via activation of hepatic p38 MAPK-dependent HO-1 pathway in a rodent model of trauma-hemorrhagic shock. However, some reports suggest that p38 MAPK activation is essential to induce organ dysfunction following hemorrhagic shock [29]–[32]. The role of p38 MAPK remains unclear. Further study is needed to evaluate the precise role of p38 MAPK. It would be more convincing if the knockout animal or siRNA is introduced to future studies.

In conclusion, the results of this study indicate that tropisetron administration alleviates hepatic injury and pro-inflammatory mediator production after trauma-hemorrhage. The decrease in hepatic injury after tropisetron treatment is likely due to a reduction in hepatic neutrophil accumulation associated with down-regulation of CINC-1, CINC-3, ICAM-1, IL-6, TNF-α, and MIP-1α. Inhibition of hepatic pro-inflammatory mediators production by tropisetron appears to contribute to the decrease in hepatic expression of chemokines and adhesion molecules. Blockade of p38 MAPK activation or HO-1 expression and the associated deterioration of other parameters suggest that the reduction of inflammation in the liver is partially mediated via a p38 MAPK dependent HO-1 pathway. Although the precise mechanism of the salutary effects of tropisetron in attenuating hepatic injury after trauma-hemorrhage remains unclear, our studies provides evidence that the p38 MAPK-dependent up-regulation of HO-1 may be critical in tropisetron-mediated hepatoprotection after trauma-hemorrhage. These findings also have implications for the potential use of tropisetron as a clinical adjunct for trauma-hemorrhage treatment.
